# Experiences of self‐care during the COVID‐19 pandemic among individuals with rheumatoid arthritis: A qualitative study

**DOI:** 10.1111/hex.13341

**Published:** 2021-08-17

**Authors:** Jenny Leese, Catherine L. Backman, Jasmin K. Ma, Cheryl Koehn, Alison M. Hoens, Kelly English, Eileen Davidson, Shanon McQuitty, James Gavin, Jo Adams, Stephanie Therrien, Linda C. Li

**Affiliations:** ^1^ Department of Physical Therapy University of British Columbia Vancouver British Columbia Canada; ^2^ Arthritis Research Canada Vancouver British Columbia Canada; ^3^ Department of Occupational Science and Occupational Therapy University of British Columbia Vancouver British Columbia Canada; ^4^ Arthritis Consumer Experts Vancouver British Columbia Canada; ^5^ School of Health Sciences University of Southampton Southampton UK

**Keywords:** coping, COVID‐19, patient and public involvement, rheumatoid arthritis, self‐management

## Abstract

**Objectives:**

This study aimed to explore the impact of the coronavirus disease 2019 (COVID‐19) pandemic on self‐care of individuals living with rheumatoid arthritis (RA).

**Methods:**

Guided by a constructivist, qualitative design, we conducted one‐to‐one in‐depth telephone interviews between March and October 2020 with participants with RA purposively sampled for maximum variation in age, sex and education, who were participating in one of two ongoing randomized‐controlled trials. An inductive, reflexive thematic analysis approach was used.

**Results:**

Twenty‐six participants (aged 27–73 years; 23 females) in British Columbia, Canada were interviewed. We identified three themes: (1) *Adapting to maintain self‐care* describes how participants took measures to continue self‐care activities while preventing virus transmissions. While spending more time at home, some participants reported improved self‐care. (2) *Managing emotions* describes resilience‐building strategies such as keeping perspective, positive reframing and avoiding negative thoughts. Participants described both letting go and maintaining a sense of control to accommodate difficulties and emotional responses. (3) *Changing communication with health professionals* outlined positive experiences of remote consultations with health professionals, particularly if good relationships had been established prepandemic.

**Conclusion:**

The insights gained may inform clinicians and researchers on ways to support the self‐care strategies of individuals with RA and other chronic illnesses during and after the COVID‐19 pandemic. The findings reveal opportunities to further examine remote consultations to optimize patient engagement and care.

**Patient or Public Contribution:**

This project is jointly designed and conducted with patient partners in British Columbia, Canada. Patient partners across the United Kingdom also played in a key role in providing interpretations of themes during data analysis.

## 1 INTRODUCTION

1.1

Self‐management, or ‘self‐care’ (a term preferred by some individuals living with rheumatoid arthritis [RA]), is a key component of successful chronic illness management.^1^ Broadly, self‐care refers to individuals actively choosing and implementing strategies to reduce their symptoms and live as well as they can with their illness. For people with RA, this involves managing their medications, being physically active, eating well and seeking medical help if their arthritis flares.^2^, ^3^

On 11 March 2020, the World Health Organization declared coronavirus disease 2019 (COVID‐19) a pandemic, posing new challenges for people with RA to manage their self‐care.^4^ For instance, fear from the increased likelihood of testing positive for COVID‐19 as a person using immunosuppressive treatment may lead to avoiding grocery shopping, walking outdoors or pharmacy visits to reduce exposure in public spaces.^5^ With rheumatology practices and rehabilitation services transitioning to telehealth consultations or cancelling appointments, there may also be changes to accessing health professionals.^6^, ^7^ Some individuals with RA may experience administrative burden in arranging consultations or avoid seeking medical attention for new or worsening health problems. Gyms and swimming pools were closed in many communities during the pandemic, reducing access to exercise facilities. Furthermore, some worried about access to essential medications, such as hydroxychloroquine.^6^ Taken together, these challenges may have significant impact on individuals' self‐care practices and their physical and mental health in many countries impacted by the pandemic.

Underpinned by a constructivist paradigm and applying reflexive thematic analysis, this qualitative study explored in depth the impact of the pandemic on self‐care from the perspectives of people living with RA. Specifically, we focused on individuals' experiences in managing stress, staying active, eating well and accessing necessary medications during the global health crisis.

## 2 PATIENTS AND METHODS

This study was situated within two randomized‐controlled trials (RCTs) on self‐care interventions involving the use of a wearable activity tracker, a web‐based app and activity counselling with a physiotherapist in Canada.^8^, ^9^ At the end of the RCT interventions, participants were invited to share their experiences in an interview related to the effects of the interventions on their self‐care.

To be eligible for the RCTs, participants needed to have: (1) a physician‐confirmed diagnosis of RA, (2) not undergone joint surgery in the past 6 months, (3) no history of acute injury to any joints in the past 6 months, (4) an email address and daily access to a computer or mobile device and (5) no contraindications to be physically active as identified by the Physical Activity Readiness Questionnaire.^10^ Recruitment occurred at rheumatology clinics in the province of British Columbia (BC), Canada, and through patient groups, Facebook and Twitter. RCT participants were purposively sampled for the present study to achieve maximum variation in demographic characteristics (age, sex, education). Participants also needed to have reached the end of the RCT intervention to be interviewed between March and October 2020, coinciding with Phases 1–3 of the province's pandemic response plan (File S1).^11^

All interviews were conducted by phone by a PhD researcher with qualitative research experience (J. L.) and focused on two topics: (1) participants' experiences with the intervention (an original study goal) and (2) impact of the COVID‐19 pandemic on self‐care (a goal added as a result of the pandemic occurring while the RCTs were underway; File S2: Interview guide). The researchers (L. L., C. B., J. L.) initially created the semi‐structured interview guide and, in consultation with five patient partners (C. K., A. M. H., K. E., E. D., S. M.), modified it before interviews began and periodically throughout the study to add content relevant to exploring the impact of COVID‐19 on participants.^11^ The RCTs and addition of COVID‐19‐related questions were approved by the University of British Columbia Clinical Research Ethics Board (H15‐01843, H17‐03424). Written consent was obtained from all participants. Participants chose their own pseudonyms.

### 2.1 Data analysis

Audio recordings of interviews were transcribed verbatim and transcripts were deidentified. Data analysis was informed by a collaborative and reflexive thematic analysis approach within a constructivist paradigm, which aligns well with our research question as it is suitable for developing an understanding of multiple realities that are socially and experientially based.^12^, ^13^, ^14^, ^15^ Two researchers (J. L., J. M.) independently read and reread a sample of three interview transcripts, making notes on points of interest shaped by the research question as well as broader questions around what was going on in the data, before meeting to share and reflect on each other's perspectives. They next independently generated semantic codes inductively from this sample and continued to make notes of points of interest and to question and query assumptions made in interpreting and coding the data. They then met regularly to collaboratively rework, discard or create new codes. The researchers next sorted codes to construct the most meaningful candidate themes through discussion. These candidate themes were applied to subsequent transcripts, with researchers modifying, rejecting or creating new themes or subthemes as directed by their engagement with these transcripts. Meetings were held with patient partners (A. M. H., K. E., E. D., S. M.) to discuss whether candidate themes resonated with their experience. The researchers re‐visited previously coded text to check how newly gained perspectives of patient partners verified, modified or refuted candidate themes. The researchers next defined each theme and subtheme by working out its scope and central organizing concept, and consulted with the full team to check coherence. The BC Centre for Disease Control COVID‐19 Language Guide was consulted to ensure that inclusive language was used to label and define the themes identified.^16^ Representative quotes for each theme were identified to illustrate the experiences of the participants. Our sampling and analysis ceased upon agreement among the research team that sufficient depth of understanding was reached to address the research question. This kind of interpretive judgement is better aligned to our qualitative approach than the notion of saturation.^17^, ^18^, ^19^

To examine and strengthen the transferability of the findings, researchers in Canada partnered with researchers in the United Kingdom and shared the candidate themes with 13 UK‐based patient partners to better understand how people with RA managed in different global contexts during COVID‐19 and whether successful strategies may be transferrable and could be shared more widely. The candidate themes served as a stimulus for discussion, facilitated by a health sciences researcher (J. G.) in the United Kingdom, as the patient partners reflected on whether themes resonated with their own experiences of managing their self‐care activities while living with RA in the United Kingdom during COVID‐19. Detailed notes were taken by the researcher during discussions to document similarities, differences and additional experiences.

### 2.2 Patient involvement

We use the term ‘patient partner’ to describe patients, their caregivers or the public who engage in research activities in partnership with researchers. Engagement is broadly understood to occur when patient partners are actively involved in governance, priority setting, conducting research or knowledge translation activities.^19^, ^20^ Patient partners in Canada helped refine the research objective and modify the interview guide. Patient partners in Canada and the United Kingdom (all of whom live with RA) played a central role by providing interpretations of themes during data analysis. During dissemination, they are coauthors in publications and will copresent results at scientific meetings, public forums and social media. Details of patient partner involvement are reported in File S3.^21^, ^22^

## 3 RESULTS

From 16 March to 2 October 2020, telephone interviews (30–60 min) were conducted with 26 (79%) of 33 eligible participants. Of the seven (21%) who were not interviewed, one declined due to illness and six did not respond to the invitation to participate. Seven (27%) interviews took place during Phase 1 of the province's pandemic response plan, six (23%) took place during Phase 2 and 11 (42%) took place during Phase 3. Participants were 27–73 years old (with a median age of 54 years), and 23 (88%) were female (Table 1), which is fairly consistent with the broader Canadian population living with RA.^23^ Fourteen participants (54%) were employed and two participants (8%) were volunteers. The annual household income ranged from $12,001 to over $100,000, and 18 participants (69%) held a university degree or trade certificate. Two (8%) participants lived alone. Our analysis identified three themes: (1) *Adapting to maintain self‐care* (including three subthemes); (2) *Managing emotions*; and (3) *Changing communication with health professionals*. Supplemental data to illustrate each theme are presented in Appendix I.

**Table 1:** Participant characteristics

Participant; interview date	Age	Sex	University degree or trade certificate (yes/no)	Annual household income	Employment status	Living alone (yes/no)	Phase of the British Columbia restart plan^a^
Riley; 13 March 2020	57	Female	No	$24,001–40,000	Retired	No	1
Nikki; 16 March 2020	33	Female	Yes	$12,001–24,000	Freelance	No	1
Anastasia; interviewed 25 March 2020	69	Female	Yes	Over $100,000	Retired	No	1
Leia;14 April 2020	41	Female	Yes	$80,001–100,000	Part‐time employee	No	1
Heather; 22 April 2020	45	Female	Yes	Over $100,000	Household work	No	1
Nic; 23 April 2020	33	Female	No	$60,001–80,000	Household work	No	1
Sarah; 27 April 2020	61	Female	Yes	$40,001–60,000	Part‐time employee	No	1
Betty; 25 May 2020	27	Female	Yes	Over $100,000	Full‐time employee	No	2
Linda; 26 May 2020	51	Female	No	$80,001–100,000	Full‐time employee	No	2
Sally; 27 May 2020	54	Female	No	Over $100,000	Full‐time employee	No	2
Margaret; 28 May 2020	58	Female	Yes	$60,001–80,000	Household work	No	2
Mary; 4 June 2020	36	Female	No	$60,001–80,000	Household work	No	2
Carol; 16 June 2020	38	Female	Yes	Over $100,000	Full‐time employee	No	2
Betty; 25 May 2020	27	Female	Yes	Over $100,000	Full‐time employee	No	2
Chris;25 June 2020	66	Male	Yes	Over $100,000	Retired	No	3
Vanessa; 2 July 2020	45	Female	Yes	$60,001–80,000	Full‐time employee	Yes	3
Penelope; 3 July, 2020	60	Female	No	Prefer not to answer	Volunteer	No	3
Marilyn; 3 July 2020	31	Female	Yes	$60,001–80,000	Full‐time Employee	Yes	3
Victoria; 14 July 2020	29	Female	Yes	Prefer not to answer	Full‐time employee	No	3
April; 14 July 2020	61	Female	Yes	$60,001–80,000	Self‐employed	No	3
Daff; 7 August 2020	54	Female	Yes	$60,001–80,000	Volunteer	No	3
Walt; 12 August 2020	73	Male	No	$80,001–100,000	Retired	No	3
Mark; 30 August 2020	67	Male	No	$24,001–40,000	Full‐time employee	No	3
Dr Pooh; 4 September 2020	73	Female	Yes	Over $100,000	Retired	No	3
Danielle; 29 September 2020	61	Female	Yes	Over $100,000	Retired	No	3
Ruth; 29 September 2020	65	Female	Yes	$60,001–80,000	Self‐employed	No	3
Susana; 2 October 2020	43	Female	No	$80,001–100,000	Household work	No	3

^a^ The BC Restart Plan adopts a phased approach to mitigate the impacts of COVID‐19. Each phase provides guidance for individuals. Phase 1 ended on 18 May 2020. Phase 2 ended on 23 June 2020. Phase 3 was ongoing until the phased approach was put on pause by province‐wide restrictions coming into effect by order of the Provincial Health Officer on 19 November 2020 at midnight. Further details can be found at https://www2.gov.bc.ca/gov/content/safety/emergency-preparedness-response-recovery/covid-19-provincial-support/bc-restart-plan

### 3.1 Theme 1: Adapting to maintain self‐care

Participants described how routine activities necessary to self‐care (e.g., being physically active; getting groceries) were disrupted across all pandemic phases (Table 2, Quotes 1–2). For example, in Phase 2, Carol stated ‘early on into the pandemic… my husband really stepped up. He did all the shopping and all the errands we had to run… It was just having interaction with people, that really worried me’. Many avoided public facilities (e.g., gyms) because they wanted to prevent exposure to the virus. Nikki explained in Phase 1 ‘falling off my routine now is meaning I'm getting tireder, my pain's increasing, I'm not being as active’. Carol reported in Phase 2 ‘I'm not nearly as active as I was last year at this time’. In Phase 3, Daff intended to avoid going to the gym and curling for a while ‘because that is also an inside place and way too many people’.

**Table 2:** Illustrative data for Theme 1: Adapting to maintain self‐care

1_Riley (Phase 1): Like I said, like I have been going over to the mall but now I don't want to go over to the mall because of this virus, right? [Laughter] And so kind of limited, you know, in that way at the moment.
2_Dr. Pooh (Phase 3): We go to the Rec Centre and that's just beginning to open. The same with Aquafit at the wave pool, it's just now opening up… I'm not going to do it because this being a suppressed immune system, my doctors are advising that I don't… I'm not going to risk my health for my health. [Laughs] So, it's kind of a real conundrum there, isn't it? …a lot of those things that I was doing before… I couldn't do when the pandemic came along… my normal activities were very curtailed…
3_Penelope (Phase 3): When I did go out for walks, I had to—my husband and I first drove around in the car to look for areas that have wide streets and very few people walking. You know, you would find—I mean, obviously you would find somebody walking and some walking the dog and stuff, which is normal. However, not crowded… So even though it's lovely, we've had to explore areas that maybe were not as well‐used by other people, to just be able to do the social distancing more efficiently, right.
4_Marilyn (Phase 3): The dog parks closed, so [laughs] you know, my walks had to be rostered and the trails got so busy… sometimes people are not very—they're not very aware of maybe how close they stand to you. So it kind of—it was not enjoyable. I had to get looking for other places to take my dog, instead of my usual trail. Because you know, it's hard for people to get used to their social distancing, right, and I do get that. But then, a lot of people started riding bikes as well, so the trail was very busy. I just started to actually take time that I felt weren't going to be so busy… it's so awkward to tell people, because you just don't know how they'll take it… I do remember actually the one time a girl tried to ride past me and my dog got scared and she literally right beside me, and I dropped what I had in my hand, and I got—but again, I didn't say anything. Because you just don't know if someone will react negatively towards that, and I don't want confrontation… The signs are everywhere and people—you know, some people are just not going to listen and I'd rather not get into it with someone.
5_Chris (Phase 3): I had to renew my prescription about two weeks ago and there was a shortage. They, you know, were basically said, you know, like kind of had to buy something that was twice as big and only use half of what was there because there was no preservative in it. But anyway it only took a day or two and they found a source of this, so they gave me enough for four weeks. And usually they give me enough for 10 weeks. I was a bit annoyed at having to pay for two weeks and only use one. But I mean with everything else that's going on in the world that's a minor problem.
6_Penelope (Phase 3): Everything that I volunteer with stopped, came to a screeching halt. And so it's of course, again, most of the learning that you've had to do with new programs… via Zoom programs or, you know, webinars or whatever. Like, on a platform where you're supposed to be maybe sitting at times. I do get up and stretch, and I do my shoulder—you know, I do a little bit of stretching and stuff when I'm doing that. But the point is, it took a turn in a different way, where you could be physically active, because you walk to a venue.
7_Linda (Phase 2): I think the difference now is because I don't go to the office… So, I do think for that, my own wellbeing, my whole health benefit has improved significantly… at home I have a lot more flexibility… I'm not doing that two hour commute anymore, so it's saving me time. I'm able to go out for a fresh walk at lunch or—and after work, if we have the time… I'd try to do a workout at least three, four times a week, but that's it. Now I'm doing it every day, sometimes twice a day.
8_Danielle (Phase 3): I've probably been more vigilant about eating healthier… I'm doing a lot more creative cooking, shall I say… I have a special interest in diet and anti‐inflammatory diets and RA, like that's always been an interest of mine. So I had been pursuing and taking some little mini online workshops… So that's an interest of mine, so having the time, the pandemic has given me the time to kind of carry that to the next level and see what I can do with it.
9_Susana (Phase 3): …With my daughter not going to daycare I didn't have to do any commuting so we would just go for walks. So it just started out with lots of walking and then teaching her to bike… So, the start of this pandemic it actually really jumped me forward into my activities.

#### 3.1.1 Subtheme: Taking added measures to prevent virus transmission

Participants described how they were able to adapt to maintain their self‐care whilst preventing virus transmissions and the challenges that arose when making adaptations. Ruth stated ‘As long as I wear a mask and I have some hand sanitizer with me in the car and in my purse… If I have to go into a store, I always maintain my distance… I always wash and sanitize my hands when I go in the store… then I go in my car, and I sanitize again… I come home and I do it again, just to make sure’. Added measures to prevent COVID‐19 transmission also included wearing a mask and finding quieter routes to walk or run that were better suited to physical distancing measures (Table 2: Quotes 3–4). During Phase 2, Penelope returned to grocery shopping during hours designated for individuals at higher risk of becoming seriously ill if they tested positive for COVID‐19, finding it ‘exhausting’ to practice protective measures. Participants sometimes experienced unease with protective measures, feeling uncomfortable or scared in a public space, as not everyone followed physical distancing measures (Table 2, Quote 4).

#### 3.1.2 Subtheme: Adapting to changes in medication access

The majority of the participants did not experience disruptions accessing medication; however, some were forced to adapt to changes, impacting their continued access (Table 2, Quote 5). Leia experienced ‘really high anxiety’ during Phase 1 when she could not refill her biologic medication through her usual pharmacy because they were ‘too busy right now to be dealing with specialty stuff’. After many phone calls with her extended health insurer, she felt ‘utter relief’ when another specialty pharmacy shipped the medication at no extra charge. Nic also reported in Phase 1 that her pharmacist could ‘only give me a three‐month supply at a time because (hydroxychloroquine) is in short supply and they gave it me from a different supplier that I've never had before’.

#### 3.1.3 Subtheme: Improving self‐care while spending more time at home

Changes in participants' employment or volunteer work brought about by the pandemic also affected their choices about physical activity (Table 2, Quote 6). Carol ‘was missing a lot of steps from just not being at work’ and described how in ‘the first little while I wasn't doing a whole lot. There was a lot of sitting’ before feeling inspired to ‘think outside the box. So I started doing stair climbs in my building. Anything I could do without actually going out’.

Being laid off or working from home led some participants to have more energy or be more physically active than they were prepandemic (Table 2, Quote 7). Leia found that not going to work had helped ‘my body to rest… the typical energy draining of everyday life isn't a factor’. Having closed his business, Mark took a new job in home delivery services, recounting ‘I'm down 30, 35 pounds from where I was… it's all the walking’. Betty reported ‘it's a lot easier to sort and plan and prep healthier meals’ since working from home. Vanessa reported, since being laid off, ‘I've been biking, I've been canoeing… I think it's better. By not having an excuse of, “Oh, I'm a little bit tired, maybe I'll do the workout tomorrow, from work’”.

Retirees and homemakers also experienced self‐care improvements (Table 2, Quotes 8–9). With her husband working from home and her daughter home from school, Heather, a full‐time homemaker, remarked in Phase 1 ‘our only real change has been to go for a walk… that's actually I think improved my activity’. Chris, a retiree, commented in Phase 3 that ‘my eating habits probably improved slightly because of more home cooked meals and fewer fast food and fewer eating out meals’.

### 3.2 Theme 2: Managing emotions

Many participants experienced stress, anxiety or fear, commonly prompted when leaving the home (Table 3, Quote 1) through concern for family members who were often vulnerable or high risk (Table 3, Quote 2), by changes in employment status and finances (Table 3, Quotes 2–3) or unknowns related to medication access or adapting to new protective measures (Table 3, Quotes 4–5). However, some experienced less stress during the pandemic than pre‐COVID‐19. For example, Linda reported ‘less stress in my personal life… just being able to work from home’. Anticipating that she and her husband would not be able to work after the pandemic was announced, Betty also stated ‘I was able to prepare… we did all these things to cut our expenses before our incomes were cut… I feel like today I can breathe easy. I'm actually significantly less stressed than probably before all of this’.

**Table 3:** Illustrative data for Theme 2: Managing emotions

1_Sally (Phase 2): I started to feel like sort of mild anxiety at the beginning of this COVID situation… Maybe a tightening in my stomach and just like unease maybe. Mostly when I would go to the grocery store. Because it looked so different as far as—First it was just so different an experience for us and just seeing all those empty shelves, so I felt a little anxious about that. And I was completely aware of why I was feeling it and knowing that's just a reaction to the situation. But it was just very odd, like just such a big change in our life.
2_Betty (Phase 2): That was the only thing at first that was stressful to me, because my parents are in sort of a high‐risk age category. My dad is 77 and my mom is 67 and—No, is my dad 78? Anyways, he's older. So, I was definitely worried for them at first… in mid‐March when everything started to happen I kind of did the pre‐worrying where I was like “Oh my gosh, we're not going to be able to work, we're going to have all of this financial whatever” and I was worrying about my parents… I did go through probably one or two weeks of like fairly intense stress before my husband not being able to work and my hours being reduced… I've been able to take it in stride because we came up with a plan to sort of be able to handle it.
3_Vanessa (Phase 3): I am so sad about work… that was like really my dream job. They decided to shut down the Vancouver operation, so they laid off 12 of us in the team… I lost my awesome job, but I believe there's another opportunity, it will come… because of the financial commitment of paying my mortgage and paying so many things. So at the beginning it was like, ‘Oh wow, what am I going to do?’ Yeah, it created a little bit of stress… but then I relaxed because we have a wonderful government that is helping us… I am sure I will have another awesome job, it's just a matter of time.
4_Nic (Phase 1): It's a little terrifying because it's one of the things that Donald Trump decided was going to cure COVID [Laughter] so it's a little hard to come by right now. …it's woken me up a few times in the night. Am I going to be able to continue taking this medication that, like, essentially enables me to live a normal life… Talking to my therapist helps because she reminds me to do things like practicing being mindful, what can I control, …so I have three months of my prescription. So instead of worrying about, in four months from now, just making a plan to refill my prescription earlier knowing that it's going to be a little bit more complicated, probably, and to not borrow tomorrow's troubles. …I don't need to give into worry, and it is a good tactic for me. Like, I don't know if everybody is the same but I know when I wake up in the middle of the night being a little panicky, like, and anxious, is it going to be OK, I can think about what I have done, not about what I can't control but what I can control… There's no point in spiralling into worried worries. There's enough worries… there's definitely a little bit of fear and anxiety there with how this is going to impact my life. I mean, you can only plan so far in advance.
5_Danielle (Phase 3): Well, there certainly was a huge amount of stress at the beginning when no one really knew anything and my husband essential service and he was out there and I was here protecting myself and then he'd come home. And so, there was a lot from my end on, I don't know how to deal with it. I don't know if you're supposed to stay away, and do I have to disinfect every minute? That kind of thing at the beginning, and obviously that changes over time and we still take precautions, but it was nothing like it was at the beginning… So, it's definitely been a learning curve and a calming curve, I guess. I mean, kind of wrap my head around what I can control and what I can't.
6_April (Phase 3): I think [provincial health officer] is doing an excellent job. I don't think we'd be as good as we are if [she] wasn't there. I think the thing that I get out of her the most is she has such a calming voice when she's doing something, it's such a calming voice that she has.
7_Vanessa (Phase 3): Well, I'm 47, right. So I think the older you get, the wiser you get, in the way that when difficult situations or challenges happen, that it's kind of normal with life. I think it came from the arthritis because once you have been in pain… that's when you realize, well I don't have health I don't have anything… That's how I learned to kind of shift my mind.
8_Marilyn (Phase 3): I was, like, oh no, because at that time there was a lot of Hydroxychloroquine being one of the pills that they were looking into, to see if maybe it could help with COVID treatment. And so I thought, you know, when you see in the media, and then I asked the pharmacist and he was, like, oh yeah, I kind of read something about that. So I was … like, oh‐oh, what's going to happen if I can't get it when I need it? So I was a little bit scared… I kind of just took it in stride and just was, like, okay, well, he said it would be a few days, so just worry about it then, if you don't get it in a few days.
9_Ruth (Phase 3): This is the reality of it and people better get used to it. And I mean, it is what it is. And we can't change the way it is… So, we better get used to living like this for some time yet, because that's just the reality of it and the facts. And so, you know we have to adjust. It's just like anything else, right… It could be worse… that's how people have to look at it… Like, I think you have to keep a positive attitude, that's what I think… I think it's really important. I really do.
10_Leia (Phase 1): The realisation I've come to is that I know I'm not alone, I'm not the only one that's going through it… this is the thing that I find has become an amazing positive throughout all of this crazy… And then Miranda [Hart] wrote [on Twitter], ‘This is a really good way for people to understand… As well as dealing with illness there is daily grief on missing out on the simplest things people take for granted; a café, a walk and bus ride and views’ and she went on… it's just it beautifully articulates how hard life with chronic illness is. And that it is a grieving process. And it's hard. And usually, the responses we get aren't filled with empathy and understanding like that.

Participants commonly highlighted that the government's response eased their anxieties (Table 3, Quote 6). Betty recalled ‘the fact that our government has like kicked in with reassuring people so quickly and we have such good health leaders here, like my confidence in them has significantly helped reduce the stress’. Marilyn, however, highlighted that she ‘did need a break from hearing non‐stop updates… It was, like overwhelming’.

A variety of resilience‐building strategies were described across participants to deal with emotions during the pandemic. These strategies included keeping perspective, letting go and maintaining a sense of control, taking difficulties in one's stride, positive reframing and avoiding threatening thoughts. Keeping perspective helped them to keep negative emotions at bay. For example, set against difficult experiences before the COVID‐19 outbreak, participants commonly viewed the impact of the pandemic on their self‐care as ‘all relative’ (Table 3, Quote 7). Margaret recounted ‘I've been in isolation because of chronic illness for years now… so, I'm ideally trained for this… my brother died in January as well… with all that to worry about COVID is no big deal’. Some highlighted how letting go of the ‘uncontrollable’ and focusing on ‘what can I control’ helped (Table 3, Quotes 4–5). For example, when feeling ‘a little panicky’ about possible interruptions in her access to medication, Nic stated ‘I can think about what I have done, not about what I can't control but what I can control’. Taking difficult circumstances ‘in stride’ or going ‘with the flow’ also helped some participants to manage their emotions (Table 3, Quotes 2, 9). Chris reflected ‘our adult children, they were all off their heads. They were all, oh my gosh you can't go out, we'll buy you this and you stay isolated, and don't you do this and don't you do that. So, we were chuckling at them, but at the same time we were being conscientious… we just kind of went with the flow’.

Participants also kept perspective by keeping a positive attitude in difficult experiences (Table 3, Quote 9). For example, while Betty and Vanessa felt anxious about changes to their employment, both found positive meaning in their experience, commenting ‘it's all stressful… I guess what I feel about it is there's no price I wouldn't pay to make sure that my family and friends are safe’ or ‘I am sure I will have another awesome job, it's just a matter of time’. Others stated that a new sense of collectiveness was an ‘amazing positive’ of their pandemic experience (Table 3, Quotes 10). Penelope stated ‘we're all in it together and are all looking for ways to survive it together and are all battling our challenges together… there's been such encouraging stories that have evolved from this, because people have found themselves in situations, but also so much caring and so much compassion has come out of it as well’.

Other resilience‐building strategies that supported self‐care occurred spontaneously because of pandemic restrictions. For example, Betty commented that ‘another silver lining’ was that getting better sleep and being ‘able to get a reasonable amount of activity’ had helped her be ‘less prone to getting irritated’. On days when she experienced a low mood, Penelope found that ‘being able to speak, like, even with a friend or with a family member’ or listening to music ‘lifts my spirits’.

### 3.3 Theme 3: Changing communication with health professionals

Participants interviewed in Phase 1 typically had not experienced remote consultations with health professionals when interviewed. Participants interviewed during Phases 2 and 3 described experiences of remote consultations with health professionals, commonly speaking positively of their experience (Table 4, Quote 1). Victoria felt comfortable speaking with her counsellor, occupational therapist, psychiatrist and her family doctor over the phone during the pandemic, reflecting ‘I'm actually a really big phone person so I don't mind’. Mark also felt comfortable communicating by phone with his family physician ‘a number of times for prescription renewals and such’ during the pandemic, commenting that ‘my doctor's a good guy… So either way, phone or in person, I always like to see him… He's an upfront good, communicative, friendly doctor’. Having had appointments with her rheumatologist and family physician by videoconference or phone during the pandemic, Penelope highlighted that having built good relationships with her health professionals pre‐COVID‐19 ‘made all the difference during this time’.

**Table 4:** Illustrative data for Theme 3: Changing communication with health professionals

1_Marilyn (Phase 3): I remember I had one appointment—telephone appointment—when COVID really hit at the beginning. And it was a good one, it was a good conversation. What I really like about my doctor is, he's very thorough and he really listens. …a phone conversation was enough for me to have all my questions answered and he just emailed me my bloodwork requisition… as long as it's just a check‐up and I'm feeling okay, I really don't mind. But yeah, if I was not feeling well and I wanted him to assess something, I think it would be really too bad if I couldn't have that opportunity… if I felt there was something he needed to do, like, physically, on my body, then that's when I would feel, like, that's the best… otherwise, I'm all for the phone conversation.
2_Walt (Phase 3): My rheumatologist… would generally look at my joints and spend some time talking about protecting the joints and that was usually in person, and we couldn't do that anymore, so we tried to do it over the phone… the in‐person [consultations] are much more effective, because he gets to see what he can't see over the video.
3_Dr. Pooh (Phase 3): My family doctor has—she's wonderful and she has had me getting all of my vaccines up to date… then for other things, she'll give me a call, just to talk on the phone and that's been helpful too… And I don't – I don't imagine other people are quite as lucky as I am to have someone so proactive, but then my rheumatologist, the same thing. She's doing it by phone. I haven't been in to see her, but she calls me every three months… And she says to me, I'll have to get my blood done and all that kind of stuff, that you have to do in person, so. If they were looking at a broken leg, or like I've developed a rash on my back, it's not as easy to describe it when you're talking over the phone. And …my Rheumatologist prescribed something for the rash on my back. And, you know, she didn't see me, but she did it based on what I described. So, it's probably not as exact as if she saw me, but certainly it's quite functional.

Participants' willingness to engage in remote consultations appeared to be shaped by their relationship with the health professionals. For instance, Mary remarked that while she felt comfortable having an appointment by phone with her family doctor of 13 years who she knew ‘so well’, she expected that ‘it would be more awkward with my rheumatologist’ if she were to have an appointment remotely with him because ‘I have only seen him a few times… he's a very brisk person’. Danielle had a remote consultation with a health professional she had not met and found it ‘a little bit weird… when you meet someone in person you sort of… form your impression… I felt Zoom somewhat made that artificial… I didn't get a true sense of him as a person’.

Participants reported benefits from remote appointments. Mark remarked ‘it's easier to get hold of them on the phone than it is to make an appointment… my family doctor is booked up weeks sometimes in advance’. Penelope mentioned ‘even outside of COVID, there are some days that just even getting transportation or getting on transit… to and from your health professional's office, is a challenge’. Others, however, reported challenges when attending appointments remotely, highlighting for example that in‐person consultations are ‘more effective’ if a physical examination was needed during the appointment (Table 4, Quotes 1–3). In Phase 2, Carol stated ‘I just had one phone call with [my rheumatologist]… It really wasn't much different than the fact that I just wasn't seeing him… But I think in future it would be nice to just actually have him look at a few problem joints… Because it does happen sometimes where I think everything feels good and he'll go, “Well what about this one?” I'm like, “I didn't feel anything”, he goes, “Well it's swollen” and I'm like oh, I guess I'm used to it’.

Three participants described in‐person interactions that they had with health professionals in Phases 2 or 3 of the pandemic. During an appointment with her specialist, when he had requested a physical examination, Ruth felt ‘perfectly safe’ as physical distancing protocols were followed, and personal protective equipment (PPE) and hand sanitizers were used. She reported that she had felt less safe during a recent hospital visit, where it had been hard to maintain physical distancing with ‘so many people’. Dr. Pooh also found it reassuring that her family doctor wore a mask and a visor during an in‐person visit. However, Carol attended her infusion clinic and commented that her in‐person interaction with the nurses was ‘not as personal as it used to be. You can't see their faces… They're a lot busier now, like having to like put on the PPE, take off the PPE’.

### 3.4 Consultations with UK‐based patient partners

Experiences of participants in Canada typically resonated during consultations with 13 patient partners in the United Kingdom, who had also experienced disruption to self‐care routines and taken measures to prevent virus transmission in adapting to maintain their self‐care. Some also found that aspects of their self‐care improved while spending more time at home. One patient partner, for example, participated in a physical activity initiative to encourage walking or jogging led by the National Health Service called ‘Couch to 5K’ with her son, who had returned home after the pandemic was announced.^24^ Unlike Canadian participants, many UK patient partners had groceries bought for them by family or friends for at least the first 12 weeks after the pandemic announcement. Some had received a government notification after being identified and classified as ‘clinically extremely vulnerable’ to becoming seriously ill if they tested positive for the virus, advising them to take extra steps (i.e., to self‐isolate or shield at home) to avoid virus transmission.^25^ It was advised that they not leave their homes and minimize all nonessential contact with other members of their household, which prompted some to feel a ‘little agoraphobic’, hypervigilant, isolated, depressed or a ‘bit of a burden’ on others in the household. One patient partner felt ‘very privileged’ to have a nice home and garden to shield in, while being acutely aware of others in different situations.

Many UK patient partners related to the emotions (e.g., anxiety, fear) described in preliminary themes and, unlike our Canadian participants, some recalled feeling lonely or having thoughts of death. Also, whilst some patient partners in the United Kingdom found daily media updates to be helpful, others described government guidance reported in the media as confusing, scaremongering or untrustworthy.

All UK patient partners reported remote consultations with health professionals during the pandemic, agreeing that in‐person consultations were more effective if a physical examination was needed. Unlike Canadian participants, some UK patient partners reported negative experiences with remote consultations, feeling that they had fallen ‘through the cracks online’. These patient partners typically lived in conurbations that comprised separate towns and legislative areas.

## 4 DISCUSSION

Our study provides novel insight into the experiences of self‐care among individuals with RA during the first wave of the COVID‐19 pandemic (March–October 2020) in BC, Canada. Participants experienced disruption to many aspects of their self‐care routines. They commonly expressed how staying active, buying groceries, taking medications and continuing healthcare visits had become more challenging and complex, as they adapted to government‐advised physical distancing measures. Little is currently known about the impact of the pandemic and its response strategy on the physical activity of individuals with RA, a population already characterized by lower levels of physical activity and higher levels of sedentary behaviour than the general population.^26^, ^27^ Early evidence suggests lower levels of physical activity in a general population during periods of total lockdown with strict advisory home confinement (e.g., in the United Kingdom), but the same has not been found in areas without a total or a partial lockdown.^28^, ^29^ The latter is characterized by closures of schools, restaurants and bars, and cancellation of public meetings without strict home confinement, such as the case in BC. Our findings highlight how participants proactively adapted their physical activity to incorporate physical distancing measures, in some cases becoming more active than they had been before the pandemic. These findings may reflect a reality that some individuals with RA already have a desire and capability to continuously manage their health. They also align with sociological research that shifts away from a traditional focus in the medical literature on the need to *teach* self‐care towards understanding ways to *support* self‐care.^30^, ^31^, ^32^ The experiences described by our participants provide early empirical evidence that may inform clinicians and researchers to consider and study strategies that support individuals with chronic illnesses to adapt and maintain a healthy lifestyle during and after the pandemic.^33^

Unsurprisingly, emerging literature supports our findings that individuals with RA experienced heightened feelings of fear, anxiety or stress during the pandemic compared to before.^6^, ^34^, ^35^, ^36^, ^37^, ^38^, ^39^ In a survey of 361 respondents with rheumatologic conditions in the Bronx, the most affected borough of New York, USA, with a population disproportionately affected by COVID‐19, Maldonado et al.^37^ found that high levels of COVID‐related distress were reported. In addition to the stress of being high risk for infection, respondents experienced the stress of medication shortages (most frequently, hydroxychloroquine), and these stresses were highly associated with flares and disease activity scores.^37^ Most of our participants did not experience difficulty in medication access, but reports of ‘really high anxiety’ were shared among the few who did. Participants also generally trusted the government's handling of the pandemic and appreciated reassuring communications, although some felt overwhelmed at times by an information overload. Our findings indicate that participants generally demonstrated resilience in managing their emotional health, by using a range of strategies including positive reframing, letting go of the ‘uncontrollable’, listening to music or distanced connections with family members. Previous research has demonstrated that resilience benefits individuals with rheumatic diseases through improved physical and psychological functioning, lower fatigue and disease activity and higher mental health‐related quality of life.^40^, ^41^ In a qualitative study of 18 individuals with RA, the process through which resilience is acquired is dynamic and complex, meaning that while a person with RA may demonstrate resilience in one situation, they may struggle in another.^42^ Helping people expand their repertoire may be beneficial and the resilience‐building strategies described by our participants may therefore be of practical use for other individuals with RA or similar conditions struggling to cope during the pandemic, or for clinicians and researchers invested in supporting them.

Study participants generally had a positive experience of remote consultations with health professionals. Rheumatology care was reorganized in the province during the pandemic, from the initial suspension of nonurgent medical appointments, substituted by remote consultation, to the following months of gradual increases in selected face‐to‐face visits. In a US‐wide survey of 530 individuals with rheumatic diseases, almost half of the respondents experienced changes in access to healthcare at the start of the pandemic, with some appointments carried out via teleconferencing, but more frequently, appointments were cancelled or postponed, contributing to frustration and worry about whether to stop taking medications.^6^ While the full impact of the disruption in routine access to care is not yet known, our findings align with other studies demonstrating that telehealth was acceptable to individuals with rheumatic diseases in the early phase of COVID‐19 if their illness was well controlled and consultations took place with a health professional who knew their case well.^5^, ^43^ This lesson suggests telehealth as an adjunct to in‐person visits, a substitution for some but not all visits, to support individuals with RA in maintaining their self‐care. Further research could explore the role of telehealth in achieving pandemic preparedness for the future and in routine rheumatology care post‐COVID‐19. Advancing understanding of the range of patient preferences is also essential in exploring this role of telehealth, as preferences are likely to differ depending highly on the context (e.g., rapport with the health professional, whether the illness is well controlled, if a caregiver offers support for telephone communications, etc).

This study has two main limitations related to its sample. Future work should examine the impact of the pandemic among populations at the intersection of poverty, racialization, illness and other social determinants, who are known to have poorer health outcomes and are not reflected in our sample. It is also known that individuals using telehealth in the United States tend to have higher socioeconomic status with stable phone and internet services.^37^, ^44^ It is possible that remote consultations may widen present health inequalities, due to limited health literacy for example, which the limitations of our sample may not have identified. Our sample was recruited from self‐care intervention studies involving the use of technology and may be very unique. Consultations with patient partners in the United Kingdom, however, enabled us to reflect on the transferability of the findings and indicated that experiences generally resonated across these geographical, political and health service contexts. Also, since participants were only interviewed once, we could not observe how their experiences may have changed over time during the first wave of the pandemic, which was a turbulent period of rapid change. Our findings may have been influenced by social pressures to stay positive in the face of adversity, possibly making participants reluctant to fully discuss challenges during their interview, although patient partners shaped the interview guide to encourage disclosure during interviews. To our knowledge, this is the first qualitative study to explore in depth the impact of the pandemic on the experiences of individuals with RA in Canada before the first COVID‐19 vaccine was approved for use in the general population. The insights gained will serve to inform future studies to improve self‐care support, strategies to optimize in‐person and remote care during and after the pandemic and preparations for future public health emergencies.

## CONFLICT OF INTERESTS

The authors declare that there are no conflict of interests.

## AUTHOR CONTRIBUTIONS

All authors were involved in drafting the article or reviewing it critically, and all authors approved the final version to be published. *Study conception and design*: Li, Backman, Koehn, Hoens, English, Davidson, McQuitty, Adams, Gavin, Leese. *Acquisition of data*: Leese, Thierren. *Analysis and interpretation of data*: Leese, Ma, Koehn, Hoens, English, Davidson, McQuitty, Li, Backman, Gavin, Thierren.

## Supporting information

Supporting information.

Supporting information.

Supporting information.

Supporting information.

## Data Availability

The data that support the findings of this study are available on request from the corresponding author. The data are not publicly available due to privacy or ethical restrictions.

## APPENDIX I SUPPLEMENTAL DATA

**Table:**

Supplemental data for Theme 1: Adapting to maintain self‐care
Daff (Phase 3): I can see the gym from our condo, and there are usually four or five people over there at once… it's not a huge gym… I'm just not comfortable with going in. Now that I have this online one, I probably won't go there for a while… I'm not sure about curling either, because that is also an inside place and way too many people… there's four people on a team and you've got eight people on a sheet that's only about sixteen feet wide… I don't think I want to go back to that…
Nikki (Phase 1): I haven't been able to go to the gym and it's not because they've closed the rec centres, and I think they will or they should. It's just because I can't take that risk, so I'm not doing my regular routines and falling off my routine now is meaning I'm getting tireder, my pains increasing, I'm not being as active.
Carol (Phase 2): I'm not nearly as active as I was last year at this time. And a lot of it has to do with all my races getting cancelled… I do running and I do triathlons… Around this time I'd probably be biking a lot more than I am right now.
Danielle (Phase 3): Well, I think I am less active even though I do my walks. I think overall I'm less active because I'm not running out and doing this, not doing that.
Chris (Phase 3): Oh the golf. Well again it was cancelled, like, we were in Maui the first two weeks of March. When we came back it was closed down. For only about a month. And then it opened up again. It was one of the—as a matter of fact I found out by accident that—that the golf courses never were asked to close. That a lot of places closed anyway because of whatever, you know—but our course we golf on, that was only closed for, let's say, one month. Say the month of March, a bit of April. And I've been golfing ever since… overall the pandemic hasn't affected my activity level very much. I probably did a little more walking there when I couldn't golf, I'd walk around the block a few times, or walk down to the convenience store and back and things like that. But that's about it.
Ruth (Phase 3): Well before the pandemic, I was going—I was going to the gym… when they closed it down I kind of was stuck there… and I was going swimming too, which helped a lot… But I walk… I'm a very active person. I don't sit.
Danielle (Phase 3): At the beginning I was very reluctant to do anything, like I didn't—it didn't at the very beginning. My husband would go out and get all the groceries, and you know, leave them and I'd wash them all and all that, the whole nine yards. But kind of—the only thing that I do now that's still has lingered a little is I don't always—if I go out in the car, let's say to go grocery shopping or whatever or we need to just run in a place to get something, I will often not go in… So that has lingered for sure… It was extremely difficult at the beginning. It's less difficult now, but it's certainly always a consideration, it doesn't really leave my head…
Penelope (Phase 3): About six weeks ago, I started grocery shopping. As I said, in the first while, people were bringing in stuff. I was inside. My friends would basically—for shopping, my friends would bring groceries. I would send the list, they would bring the groceries. I would e‐transfer the money to them and then would begin the total thing of, you know, disinfecting and wiping things.
Carol (Phase 2): I live right by the sea wall. So I've been avoiding that the last few months because it's just too busy and it gives me too much anxiety with all the people on there. So I mean the good thing about this whole pandemic is that it's kind of forced me into the trails and figuring out other less busy routes to take around the city. So I try to make it a goal to run at least three to four times a week. Some of them was a bit of trial and error. So where's this one going to end up? But I think like over the last couple of months, I've just really gotten to know them very well. It's kind of like I'm running and enjoying running again. It's just being in the trails, being in nature. I think I'll continue with those runs. I went the other day, it was raining outside, so the sea wall was dead. I had every opportunity to use the sea wall and not feel so stressed. But I don't know, I just felt really drawn to be back in the trails again.
Penelope (Phase 3): About six weeks ago, I started grocery shopping. As I said, in the first while, people were bringing in stuff. But then I started going out when they started doing the opening for the first hour where everything's sanitized and clean for the high risk and vulnerables. And we started going out early, getting up early, which can sometimes be a feat getting there at the store and having our lists ready and actually. I've also had to shop not only for myself. I have an eighty‐three‐year old mum, so that's hard on her, so I've had to shop for her as well. And it's quite challenging, because not everybody in the store are practicing social distancing, even the store that I have chosen to go to, they had all the shields up and they were very good about the whole protocol. But actual people sometimes are not as cognisant of that… it's exhausting and it takes a lot of time, because we follow the protocol; once we go out of the house, we have to, when we come back, the shoes—there's rain and sleet outside—then we strip down, put everything into the washing machine and we take showers and, you know, go back and then of course have to—everything that's brought in has to be done. And to me, that sometimes takes my whole day and I'm exhausted.
Daff (Phase 3): When I went to the mall… It was way too crowded. People just don't give you your six feet space… I was there to buy grocery items for my daughter, so I quickly got through that area and then I left the mall… So there are a few places where I just—yeah, get a little concerned, but most of the places I go to, I feel comfortable… we had to get some photos [at Costco]—and this gentleman, he came right up to us. Now, he had a mask, so we did not have masks, so actually for him, he should have stayed away from us. But my husband actually had to tell him to back up, because he just had no sense that he was, like, right beside us… It was, like, regular Costco fiasco whenever you went. [Laughs] So we just stopped going. I mean, it's not that important to go to Costco, we could buy stuff at other places. So yeah, that was just a little – it just makes you nervous… it puts you in a position where you're scared, I guess, is what it is. So you might say things more than you would before. I'm just thinking hypothetically, I could imagine somebody who is really scared and having somebody come up with that, them getting very confrontational and the other person being – It can become a real issue, I think, you know, depending on how it's said to the other person, you know. If you're polite, like, you know, please give me my six feet. But if you get angry and yell at them, or something, you know, I could just imagine, you could end up with a lot of arguments and fights and it could get very violent even—I don't want to give it to somebody if I have come in contact with it, right… I mean, like, my daughter is a Type 1 diabetic, so she's high risk, and I definitely wouldn't want to bring it home… I don't want to be giving it to her…
Heather (Phase 1): I've had to leave so like on grocery pick‐up, so I don't actually have to go into the store but I have to go down to the store and it's brought to my car… I actually get anxiety about leaving the house, like so my emotions are like around. There's fear of the what‐if's, like what if someone coughs on me, what I catch something, what if I bring something home to my family. I'm very… like my husband's been told by every single doctor he sees which is far too many that he's high‐risk and he needs to stay home and, you know, reduce exposure, wear a mask and wear gloves and so that definitely as the person who goes out and I don't even really go out [laughs] I feel… fear.
Leia (Phase 1): The biggest stressor I have had throughout this whole COVID thing has been getting my medications. That has been ridiculous. So my husband's extended health company, they're as helpful as can be, but they don't cover dispensing fees. And most of the pharmacies near us, their dispensing fees are like $15. And with a number of medications that I have, that adds up really quite quickly. So we've been using Costco for the last few years their dispensing fee is only $4.50, that's a huge difference when you're getting a lot of meds. But with the pandemic, you couldn't get a hold of anybody. You couldn't get through to them, they were—finally when I would talk to them, they'd be like, ‘Oh yeah, we've got everything setup’, then they'd call me back and they're like, ‘Oh something's wrong with the payment for your meds’ and I'm like OK. I literally spent, over a period of about two weeks, I am honestly not over exaggerating, 12 to 14 hours on the phone just trying to get my last Humira dose. It was a complete nightmare. So I finally got to the point where I was like, ‘OK you guys have been really helpful in the past, but you are just too busy right now to be dealing with specialty stuff like this’. Because if there's one thing in this world that will instantly put me into almost a panic attack or really high anxiety, is telling me I won't have access to my biologics on the day I'm supposed to inject it. Because my biologic has made such a difference to my everyday mobility that I'm instantly in a panic. So we finally—there's a new pharmacy that just opened up a number of months ago, just down the street from me. The pharmacist there agreed to match the Costco dispensing fee until I hit my Fair PharmaCare—that one I would have to say has probably been the biggest ordeal out of this whole COVID thing, was trying to get my meds organised. That was in conjunction—lots of phone calls between my extended health company, the AbbVie program, my representative through the AbbVie Care, the support network for Humira. And the new pharmacy and the old pharmacy I guess, so it was a lot of back and forth, back and forth, back and forth. The Specialty Pharmacy is tied to my extended health, so they will be able to direct bill to my extended health. And then AbbVie is also very familiar with that particular specialty pharmacy… it was also—but not just the happiness, it's also utter relief. OK it worked, I've got some. I'm not going to miss it. It'll be OK.
Nic (Phase 1): It's a little terrifying because it's one of the things that Donald Trump decided was going to cure COVID [Laughter] so it's a little hard to come by right now. But that's a little terrifying; it's woken me up a few times in the night. Am I going to be able to continue taking this medication that, like, essentially enables me to live a normal life… Talking to my therapist helps because she reminds me to do things like practicing being mindful, what can I control, looking at the things that are—So I have three months of my prescription. So instead of worrying about, in four months from now, just making a plan to refill my prescription earlier knowing that it's going to be a little bit more complicated, probably, and to not borrow tomorrow's troubles. Like, yes, I think about it but I don't need to obsess about it; I don't need to give into worry, and it is a good tactic for me. Like, I don't know if everybody is the same but I know when I wake up in the middle of the night being a little panicky, like, and anxious, is it going to be OK, I can think about what I have done, not about what I can't control but what I can control… There's no point in spiralling into worried worries. There's enough worries… there's definitely a little bit of fear and anxiety there with how this is going to impact my life. I mean, you can only plan so far in advance.
Carol (Phase 2): I was missing a lot of steps from just not being at work… I didn't really want to go out when we first kind of on the verge of shut down. So it was very eye opening just to see how hard and how difficult it was to get steps in. I mean my apartment is only so big… the first little while I wasn't doing a whole lot. There was a lot of sitting… So I was trying to walk around it and do laps… it kind of in a way also inspired me to kind of think outside the box. So I started doing stair climbs in my building. Anything I could do without actually going out.
Leia (Phase 1): More often than not… I come home and be exhausted and wouldn't do much around the house or with the kids. Whereas now, my clinic is closed and even if it wasn't, my doctors have me on a pretty strict ‘don't go anywhere’ routine, shall we say? It's really allowed my body to rest… overall, the not being so busy, the not running around, the not you know—because my son's karate is now online, so he does it in the living room. So I'm not running there and dropping him off and then running to drop my daughter off at skating and then you know, the typical energy draining of everyday life isn't a factor for me right now. And so I'm actually doing really well. Other people are like, ‘I can't do this anymore’, I'm like, ‘No, look how high I can lift my leg off the ground. Look my arm goes up beside my ear, what a concept’. So for me it's been good… I can sleep in in the mornings… So yes, I have a lot more energy, but I also have a lot more mental energy too, because it's not quite as draining.
Mark (Phase 3): I'm down 30, 35 pounds from where I was and so it's coming off gradually, and it's all the walking. You know I'm confident that that has, it's changed a lot of my life around, you know just that walking, for mood, for brain fog and for weight loss. And, there's a phrase for it but I can't think of it right now but it's the getting out of the van with a parcel to deliver, you know, and hustling to the front door, taking your pictures, hustling back to the van and then driving again for the intermediate—no, intermittent racing of the heart rate, you know, because I go, I really do try to hustle now.
Betty (Phase 2): It's a lot easier to sort plan and prep healthier meals, we're eating out a lot less. Our diet is just so far superior to what they are when we're just—you know, the hustle and bustle and maybe you don't always have time to grab something, to get takeout. Right now we have the time to prep good, wholesome meals… We eat plant‐based anyways, but we're eating a lot more whole food plant‐based, which is always the goal, but sometimes we're working fulltime and have all of our sports and volunteering etc. it can be difficult to not just sometimes wait for the faster options.
Vanessa (Phase 3): I've been in nature, I've been biking, I've been canoeing, and I've been dedicated to the yoga and the cardio, I think it's better. By not having an excuse of, ‘Oh, I'm a little bit tired; maybe I'll do the workout tomorrow, from work’. So I think it's better.
Heather (Phase 1): Then with COVID. And my husband's only just last week gone back to work part‐time but he's working from home. And of course my daughter is at home [laughs] from school and so part of our everyday has been to get out of the house because he's very high school for complications if any of us were to become ill so we don't go anywhere. We haven't been or I haven't been in another building for however many weeks now [laughs]. So our only real change has been to go for a walk so we've been going for walk for at least 45 minutes every day and sometimes up to an hour and half. So that's … so that's actually I think improved my activity which is kind of strange.
Chris (Phase 2): Probably my eating habits probably improved slightly because of more home cooked meals and fewer—fewer fast food and fewer eating out meals. But again, not hugely.
33_Marilyn (Phase 3): I bought an instant pot, because I wanted to make my cooking more efficient. And so, yeah, I started making a lot more meals. Like, I would cook, but not a whole lot. But with COVID, yeah, I was making my meals every day. So I felt like I was eating healthier. And because I'm, like, okay, I should really try being in the best shape that I can, eating healthy and not eating junk food. And so yeah, I think it turned into a healthy habit… because I thought, oh my gosh, if I do get sick, then I want my body to be in the best—in the healthiest way it can be if something did go down and I did get sick. I wanted a strong immune system and, like, I could have one, yeah.
Supplemental data for Theme 2: Managing emotions
Linda (Phase 2): Just less stress in my personal life… Just being able to work from home. The two hour no commute, being at home, being able to do some of my chores at lunchtime, in the morning before I go to work as opposed to, like I said before, then my whole day's gone if I don't get home until 4 o'clock to fit in the workout, to fit in dinner, to fit in laundry, to fit everything in.
Daff (Phase 3): When there was the toilet paper shortage, that was—going for everybody. And you know, my daughter was testing me at one point, she was, I've only got three rolls of toilet paper left. So then we went—I remember going to grocery stores and stores, looking for toilet paper for her, because that's the time she was self‐isolating, because she thought she had COVID—she didn't. But at the time they weren't testing everybody, so she was told to just stay home and isolate. So I was buying toilet paper for them, or even groceries or medication I had to go and drop off at their door. So yeah, it was—that time was very anxious for all of us, not to the point where it affected me physically or anything, but I was definitely more anxious than I had been in a long time.
Mary (Phase 3): For a while it was very stressful to do things like go to the grocery store or go into London Drugs. I still avoid it, I still try and go less than once a week, but I go to smaller stores, because I find sometimes there won't be anybody else there… At first it was very stressful… I definitely am aware of what I'm doing it those places, so I don't feel unsafe.
Penelope (Phase 3): In it all, emotionally, obviously there have been—you go through the stages of difficult emotions and I'll say there have been times—I would say that there's only two times I've had anxiety… I basically know what to do to get things under control and to manage that. Even then, I would say that I have some days, especially when it was weather‐related‐wise, where it was low mood. And that was, you know, like, you still have those days… you find ways of doing things that would lift your spirits indoors. And I think part for me, because I'm so social, is being connected to people and being able to speak, like, even with a friend or with a family member, and hear how they're doing… I love listening to music, so I put on good music and that lifts my spirit, that's for me personally. I also like – so music is one of the medias that I use. I've used—as I've said before, I also have been praying a lot. For me, that also has lifted my spirits… it was emotional… dealing with my eighty‐three‐year‐old mother… like, she volunteered a lot, like, almost every day—and her having to be cooped up, that was not very good for her… I think the stress of having to be able to emotionally care, because she's very demanding and aggressive, and you know, the worst comes out in people's emotions when you're at that age and you change their routines and you take things away from them, and they can't be doing what they are normally accustomed to, right.
Daff (Phase 3): I did definitely have anxiety, especially—it wasn't so much for myself, but for our children, because I was worried about their jobs… So there's a little bit of anxiety still on that part. I mean, we can help the kids at any time, but we can't help for long‐term, because that's our retirement money… we definitely saved money. I mean, we're still saving more money, just because we aren't doing as much as we normally would.
Susana (Phase 3): And now it's getting back on my mind with my daughter starting Kindergarten, it's quite a worry. Quite a worry. I feel like we're almost going to have to pull her out of school in two weeks. There's been kids sneezing—there was a parent that took their daughter to school completely sick. And my daughter said the other day that there was a boy that sneezed completely on her. She goes, Mommy I felt his snot on my hand. I'm going well great you know, so it's cold and flu season. But it makes you wonder, right, if every time you want to go and get tested; now, do we have to go and get tested every time? It's like—So it's quite stressful right now, probably for me, more than this whole start of the pandemic. It's probably more stressful now than any other time… we just recently went through a colds and all of last week I couldn't get up… It was very scary. First my daughter got it. She started school on—she went one day on Thursday and then she went Monday and Tuesday she was sick. Like the third day of school. She had a cough, a mild fever and a lot of runny nose. So we took her to Children's and she did the COVID‐19 test. So my stress level—my adrenaline goes through the roof. My cortisone—I'm on a high – I didn't sleep all Monday night. I couldn't. My brain was spinning—I was staring at her, sleeping in bed with her. My—instead of fatigue, like I said, I'm just I'm buzzing like I've had 10 cups of coffee. I'm emailing my rheumatologist. I'm like setting up everything, like I said my mind is spinning, so really high stress. In the back of my mind is fear of I'm going to get it, that's obvious, she's my kid. I slept with her all night. What is this going to mean for me, right. So I'm just like preparing if I have to go to the hospital. So it was stressful. She came back negative. By Wednesday she was completely 100 percent normal… But that stress really, really exhausted me. Friday, Saturday I was sick I couldn't get off the couch. I think—so not just having the cold but all of that stress, it was… Yeah. It was scary.
Betty (Phase 2): Obviously my husband wasn't able to see patients face‐to‐face, so. And then my hours have been reduced to 60%… because I did the pre‐thinking which caused me a little bit of stress, I wasn't at all surprised or shocked by it… I think it's all stressful and there's rarely a person that you'd come across that would say they didn't feel a bit of stress… it means that obviously we have to be a lot more careful with expenditures. On top of the mortgage… but I do feel that we're saving a lot of money by not having to go out… I guess what I feel about it is there's no price I wouldn't pay to make sure that my family and my friends are safe. So, the financial implications of this, though not great, that's like the price I pay and I would pay infinitely more if it meant keeping my family safe. So, that's kind of how I feel about it. Like, sure, it sucks—and I know I am speaking from a place of privilege when there's people who are in much worse, more dire financial situations—but it definitely sucks at a time when you're just kind of starting to feel like you're getting ahead and we're in an upward trajectory, this probably going to set us back a few years. But I would pay anything to make sure my family is safe.
Leia (Phase 1): I had just lost my job. I applied for EI but I didn't qualify for EI because I was on the medical EI last year for my surgery. So I hadn't worked enough hours. There was that new CERB program that was supposed to be coming through, but we didn't really know if I was going to qualify for that either…. So there was a lot of unknowns during that time. So as each thing kind of started to get sorted out or you felt a bit more confident in it, those anxieties would come down. They would come down. One of the big things that helps me with anxiety is my knowing, OK if I'm doing everything I can on my side of things, then I have to let go of the uncontrollables and let go of the uncontrollables. Because if you hold onto those uncontrollables, it makes life really difficult, I think it increases your anxiety and makes life difficult. So I tried my best to just go for walks with the kids. Every time I leave the house, my doctors want me wearing my mask. So we would go for walks with masks on and just try to have productive ways of letting that anxiety go as well. Sometimes it works, sometimes it didn't. Sometimes I had to have a good cry or whatever. But we got through it, I got through it.
Carol (Phase 2): And a little bit of it was fear too. Fear of trying to go beyond my four walls here… I think it was the unknown. Like not knowing how contagious it was. Was I going to get it just passing by someone or touching something? So, yeah I think it was more just not knowing, like not knowing fully what the risks were… Feeling very stressed. Lots of stress too, because I didn't know what was happening with my work… so far I'm still on payroll, not gone yet… It's the stress of the unknown… I try not to worry, I guess. Like it's tough… I guess the only good—the good thing knowing in the back of my mind is that we are unionised… that's kind of my only little kind of security I feel like I have right now.
Leia (Phase 1): The biggest stressor I have had throughout this whole COVID thing has been getting my medications. That has been ridiculous. So my husband's extended health company, they're as helpful as can be, but they don't cover dispensing fees. And most of the pharmacies near us, their dispensing fees are like $15. And with a number of medications that I have, that adds up really quite quickly. So we've been using Costco for the last few years their dispensing fee is only 4.50, that's a huge difference when you're getting a lot of meds. But with the pandemic, you couldn't get a hold of anybody. You couldn't get through to them, they were—finally when I would talk to them, they'd be like, ‘Oh yeah, we've got everything setup’, then they'd call me back and they're like, ‘Oh something's wrong with the payment for your meds’ and I'm like OK. I literally spent, over a period of about two weeks, I am honestly not over exaggerating, 12 to 14 hours on the phone just trying to get my last Humira dose. It was a complete nightmare. So I finally got to the point where I was like, ‘OK you guys have been really helpful in the past, but you are just too busy right now to be dealing with specialty stuff like this’. Because if there's one thing in this world that will instantly put me into almost a panic attack or really high anxiety, is telling me I won't have access to my biologics on the day I'm supposed to inject it. Because my biologic has made such a difference to my everyday mobility that I'm instantly in a panic. So we finally—there's a new pharmacy that just opened up a number of months ago, just down the street from me. The pharmacist there agreed to match the Costco dispensing fee until I hit my Fair PharmaCare—that one I would have to say has probably been the biggest ordeal out of this whole Covid thing, was trying to get my meds organised. That was in conjunction—lots of phone calls between my extended health company, the AbbVie program, my representative through the AbbVie Care, the support network for Humira. And the new pharmacy and the old pharmacy I guess, so it was a lot of back and forth, back and forth, back and forth. The Specialty Pharmacy is tied to my extended health, so they will be able to direct bill to my extended health. And then AbbVie is also very familiar with that particular specialty pharmacy… it was also—but not just the happiness, it's also utter relief. OK it worked, I've got some. I'm not going to miss it. It'll be OK.
Marilyn (Phase 3): So I was pretty scared. So I think the things at work were teaching on a daily basis, like, protocol with patients and PPE and, you know, and our manager's not really—. You know, they couldn't give us too many answers sometimes, and with the guidelines changing based on what was emerging for the news and health updates, it was all of it very kind of frantic for a little while. Having to also be supportive with our patients while we were ourselves were often scared… I work with cancer patients, so that was just a lot… You know, you have your moments, but then once you think about it, it's just, like, well, just reminding myself, yeah, worrying about something is not going to change anything. So just do what you need to do and then hope for the best. [Laughs]… I think it was just my way of coping… we still don't really know, you know, what's going to happen. Is there going to be a second wave, you know, and we don't know treatments yet. So I just kind of kept reminding myself, just take it a day at a time, a day at a time, and if there's a day that's very frustrating, then maybe just cry about it, rub it out and move on.
Marilyn (Phase 3): I was, like, oh no, because at that time there was a lot of Hydroxychloroquine being one of the pills that they were looking into, to see if maybe it could help with COVID treatment. And so I thought, you know, when you see in the media, and then I asked the pharmacist and he was, like, oh yeah, I kind of read something about that. So I was just being unsure, like, oh‐oh, what's going to happen if I can't get it when I need it? So I was a little bit scared… I kind of just took it in stride and just was, like, okay, well, he said it would be a few days, so just worry about it then, if you don't get it in a few days.
Betty (Phase 2): That was the only thing at first that was stressful to me, because my parents are in sort of a high‐risk age category. My dad is 77 and my mom is 67 and—No, is my dad 78? Anyways, he's older. So, I was definitely worried for them at first… in mid‐March when everything started to happen I kind of did the pre‐worrying where I was like ‘Oh my gosh, we're not going to be able to work, we're going to have all of this financial whatever’ and I was worrying about my parents… Like I know pre‐worrying is not a good thing to do, but having that kick in before everything hit, I was able to prepare. So, like we took the car insurance off our second vehicle and we did all these things to cut our expenses before our incomes were cut, which really made it so that—I feel like today I can breathe easy, I'm actually significantly less stressed than probably before all of this. But I did go through probably one or two weeks of like fairly intense stress before my husband not being able to work and my hours being reduced… I've been able to take it in stride, because we came up with a plan to sort of be able to handle it.
Mary (Phase 2): I suppose the future is always uncertain, but just right now, not knowing when things are going to go back to real normal… just not knowing what's going to happen in terms of planning for school in the fall… What's Christmas going to look like? …It's all still up in the air. So nobody really knows… I just don't really worry about it right now, just kind of staying day‐to‐day and just trying to worry about that sort of stuff, not getting to far ahead of myself… it's a little hard to think about the future right now… Honestly, I have anxiety typically. So I'm actually finding that my anxiety levels are lower overall since the pandemic started. Because actually having something to deal with and with not focusing long term or letting my brain freak out about things, has been healthy for me… overall I would say my anxiety is like half of what it normally is, because I have to just focus on now and I can't worry about the future… you can never really plan for the future. But I think just having that acknowledgement right now is helping… I guess, since the pandemic started, I haven't really had the anxiety and I've actually found my RA symptoms haven't been bad.
Betty (Phase 2): The fact that our government has like kicked in with reassuring people so quickly and we have such good health leaders here, like my confidence in them has significantly helped reduce the stress… I would say that my mental state now is really good… I think the fact that they stayed really true to just being calm about everything and not using words like—You know, at first everybody was like just ‘Call it a quarantine. Why are you putting us on lockdown?’ and like aggressive words like that. Even though the rules that they put in place were essentially like what other countries were calling lockdown without using scary terminology like that. Had they used terminology like that, I do think there would have been significantly more panic, but not like—Like there wouldn't have been, I think, any additional restrictions. So, I appreciate that they use a calm approach without using sort of panic‐inducing language. That showed a great deal of restraint and responsibility. For me, personally, I'm quite claustrophobic, so words like lockdown, I'm worried that I'm not going to be able to leave my apartment would have made me a lot more stressed. So, when they were like, no, you can go outside, go for your walks, go for your runs, get exercise, make sure you do these things for your mental health, that was really good, I thought. But they were being baited quite a lot to use those types of terms. Yeah, so I think that they did that really well and the fact that there's consistency with, and openness and transparency in terms of numbers, that kind of stuff, is really reassuring to people. And the fact that they let people who are in charge of health lead the discussion, I think was more powerful. And they took Question Period and sometimes I had to like not listen to Question Period, because the questions people ask are – you know, sometimes news is so sensational or like trying to be more dramatic. But, yeah, I just thought they did a great job and provincially and federally there was a lot of strength in leadership.
Mary (Phase 2): But we're [the family] all healthy and safe and yeah, that's really all you can ask, the way things are right now. I mean it's not great, but it could be a lot worse… for about a month, we really—a month or so was really stressful… overall we've had bad days, but overall it hasn't been that bad for me overall… Just the fact that they're getting more information. There's more studies showing that where most of the transmissions coming from, that it's coming from generally being in close contact with people indoors for longer periods… it allows you to kind of say okay, you just risk assess it more appropriately… I can go into situations and have a better understanding of what the actual risk is and what steps I can take. How to protect ourselves without having to be insane about it… I found that the BC government and the team that they have been leading the information has been really helpful and really transparent. They've been a good resource.
Dr. Pooh (Phase 3): *Interviewer: I wonder what your thoughts might be on how the government have responded to the pandemic?* Oh, I think they've been fantastic. I listen to Dr. [Henry] and—and that she's been measured, she's been truthful… I think all of our governments have been really, really good… The whole virus was new and how they're handling it, I think has been quite exceptional…
Marilyn (Phase 3): Sometimes I did need a break from hearing non‐stop updates. It was kind of too much, so I would take a break every now and then… It was, like overwhelming… everything just seems bad, very negative and just hearing about different outbreaks… and people affected by it. Like, it was just really a streak of bad news and so I'm just, okay, I need to give myself a mental break… it's okay if I go a few days without knowing… Because it really got to a point where I'd walk my dog and I would listen to the YouTube live updates and I'd be, okay, no, take this time to just listen to music or just not have anything on, and so just—it was constant… Oh, they were very helpful, because I mean, [Doctor Bonnie] she shared a lot of valuable information every day, but I am very thankful we live in British Columbia and we have someone like Doctor Bonnie to guide us through this. So I felt proud and, you know, I felt they're doing everything they can. So they were very helpful, it's just that, you know, sometimes you don't want to hear it.
Margaret (Phase 2): *How has it been for you at home since COVID?* I've been in isolation because of chronic illness for years now. Pretty much the only time I go out will be to walk or I'd go to a doctor's appointment or treatment or blood work. So I'm ideally trained for this. I'm really good at it… I had to cope with it as a necessity in the beginning, because I'd literally have a difficult time walking from one room to another, I was so ill… my brother died in January as well… and my sister died at 24, a week before I was to give her a kidney transplant… with all that, to worry about COVID is no big deal… so I guess that being in such a difficult scenario, everything else seems easy. Not easy, it's just… So yeah, a lot of trauma… But it's given me great ability to adapt you know. So, silver linings, right? What doesn't kill you makes you stronger. And a very resilient individual… it's all relative, right… I just don't let anything get to me. I just go with it. And I try not to stress out about things I have no control over. And I've gotten over the illusion that I control anything… you can't do anything about it, don't worry about it. ‘This too shall pass’ has become my favourite mantra… And it seems awful right now, that will change… it's one thing you can adapt a lot better when you're old, I think. Because you have life experience to guide you through those processes. I'm very good at adapting.
Betty (Phase 2): Actually, that's another silver lining about this whole thing, is getting a full night's sleep, like consistently. It's amazing. Which I think also really helps people manage their mood probably and also being able to get a reasonable amount of activity… I'm probably less prone to like getting irritated I would say. I'm a bit more patient probably because I have a little bit more sleep.
Dr Pooh (Phase 3): I'm usually an extremely positive, upbeat, optimistic person, that's just my personality. And there are days when it just—things just get a little bit overwhelming. But then the next day, I'm fine. [Chuckles]… It's not often. It only happens, you know maybe two or three times. And I would say it's just a mild depression. But you get—and I'm never thinking that there's no hope or anything like that. No. It's just that it gets a little bit overwhelming and it's not typical of who I am and I notice it because of that… Sometimes I'll go to sleep and when I wake up, I'm better. [Laughs]… Or go read a book and I get – I distract myself from my, ‘Oh woe is me’. [Laughs] And then when I'm—when I'm finished distracting myself, I'm usually ok…
Supplemental data for Theme 3: Changing communication with health professionals
Victoria (Phase 3): I talk to the counsellor every week, I have an occupational therapist. I talk to a psychiatrist a couple of times and I have, like, a family doctor if I need to talk to them… they're all on the phone now… It's been pretty decent to be honest. I'm actually a really big phone person so I don't mind.
Mark (Phase 3): I've seen my family physician via the phone a number of times for prescription renewals and such, but not my rheumatologist… I quite enjoyed it. I wish they would get to do that more… it's easier to get a hold of them on the phone than it is to make an appointment most times… at least my family doctor is booked up weeks sometimes in advance… I think you could probably do three or four, triple the amount of appointments on the phone than you can in person I'm betting… a lot of times it's just a matter of a conversation. You talk about a prescription renewal or a dosage or, you know this is how I'm feeling… my doctor's a good guy… So either way, phone or in person, I always like to see him but the phone is fine. There's no problems with that… He's an upfront good, communicative, friendly doctor.
Penelope (Phase 3): I did have a virtual Medeo appointment with my rheumatologist, and that was great… I was quite excited that it's there… And now people are happy to do virtual calls with their healthcare professionals, and I'm excited about that… it would have to be pre‐COVID that I did it with my rheumatologist, but then we have done one since COVID. And then I've also done one with my doctor that's a call, so that's still virtual… even outside of COVID, there are some days that just even getting transportation or getting on transit, or getting on transportation to and from your healthcare professional's office, is a challenge… it's kind of cool that you have that, where they can't see you, and they can do this and they can be helpful… it's important that you have good relationships with these people and your doctor, and communicate well with them. Because that made all the difference during this time… I do have amazing healthcare professionals… they do exemplify what they do in their roles, right. They're caring and they're for their patients… So anyways, that was my COVID healthcare professional experience… My rheumatologist also has a rheumatology nurse on staff. And so what happened was that the rheumatology nurse actually was working from home ‐ so she has an office at home—and she called me and asked, you know, all the questions… It was just that I didn't travel to get there, but I felt the quality of care was the same… I feel comfortable to be able to communicate and ask the questions that I need answers for myself… So they actually actively listen to what I have to say, and I think that makes a difference… we've built that trust over time with that… just taking those few minutes to be human and to show your caring, show your compassion—I'm saying that that's what I've had from mine.
Mary (Phase 2): I've had a couple of appointments with [my family doctor]… They were fine, one was just to get a prescription… One of them was on the phone and then the last one I did, she actually has virtual appointments set up now, so they have like a secure video chat that we use… I've been with my doctor now for, oh God, 13 years. So I'm really comfortable with her, so it was fine… I think it would be more awkward with my rheumatologist honestly. I have only seen him a few times, three I think, three or four. But with my doctor, I know her so well that it was yeah, it was fine… he's [my rheumatologist] a very brisk person. And so I just think that it would be a little bit uncomfortable to talk to him over video chat, I don't know.
Danielle (Phase 3): *Interviewer: some people have talked to me about having remote consultations with their health professionals, I wonder if that's happening with you at all?* I have done it once and that was a little bit weird… You know, I had seen pictures of him online and whatever, so when you meet someone in person you sort of get your—form your impression and whatever. I felt Zoom somewhat made that artificial and it wasn't—I didn't really get a true sense of him as a person… there's a little bit of an artificial wall there even though we're looking at each other… I would definitely like to see him in person. But as a first step, yeah it was fine. So now I know, you know, I had that, and it would be easier when I am able to do that in person…
Ruth (Phase 3): …My specialist… I think he's a pretty good specialist. I mean he's bent over backwards to try and, you know help me with trying to find a medication that's going to work. And so far, we had some complications… and he hasn't been able to come up with anything… During the pandemic, we just had to do the video calling, right… because I was having flare‐ups, he just had ordered extra stuff for me and whatever. But you know it's really—it's difficult… it's hard when you're videoing—videoing over the phone… because you're not seeing each other and you can't really look… he can't look at me and touch my joints and stuff like that to actually… he's only going by what I'm telling him. So, it does make it hard to get a correct diagnosis, right. And that's what he said. So, the last time, you know he said to me, ‘Well we, you know when you tell me—I was telling him all this stuff on the phone’. The last time he goes, ‘No, no… I have to get you to come in now because I need to see you, so that I can examine you’. It went way better when we examined me. He said he could at least see… how bad I was… I just wore my mask and, you know sanitized and everything. And he had his mask on. And we sanitized even in the office and everything. I had no issues there at all. You know, I mean the protocol was very well—you know there was social distancing between us, etc. You know and he had gloves on when he examined my fingers and looked at my feet and all that stuff… I mean I felt perfectly safe—safe with him, right. I felt safer in his office than you do when you go into a hospital… you know the hospitals, I think are just—social distancing is—is a little bit not as good… they try to but it's kind of hard because you know you've got so many people, right… it just seems like they always have extra people coming in.
Dr. Pooh (Phase 3): When I go to get my vaccines, I come in, they have someone at the door when you come in. [My family doctor] She's dressed in PPE with mask and a visor. And—to take my temperature and they ask me questions and then they take me right into the room… I find it reassuring. Because a doctor's office is—people come to a doctor's office either because they're sick or they don't want to be sick. So, I would much rather be protected from a virus that you can't see, than to have somebody with—does not have a mask on.
Carol (Phase 2): I get the Actemra done by infusion, and I really gotten to know the nurses really well there. So that's all changed now because now they're completely downed out. There's a whole process of going in and going out. So it's very different, it's not as personal as it used to be. You can't see their faces. You can't smile. They're a lot busier now, like having to like put on the PPE, take off the PPE. So it's a lot different… I really feel for them. I can't imagine having to wear that all day… they're just all a really great bunch, but yeah, it's just, it feels different… I understand why we have to wear them, so I'm OK with it.
