# Supplementary figures and images for: Experiences of self‐care during the COVID‐19 pandemic among individuals with rheumatoid arthritis: A qualitative study
Source: Health Expect. 2021 Aug 17;25(2):482–98. doi: 10.1111/hex.13341 (PMC8444741; doi:10.1111/hex.13341)

**Supplementary File 1: British Columbia’s COVID-19 Pandemic Response Timeline**

**
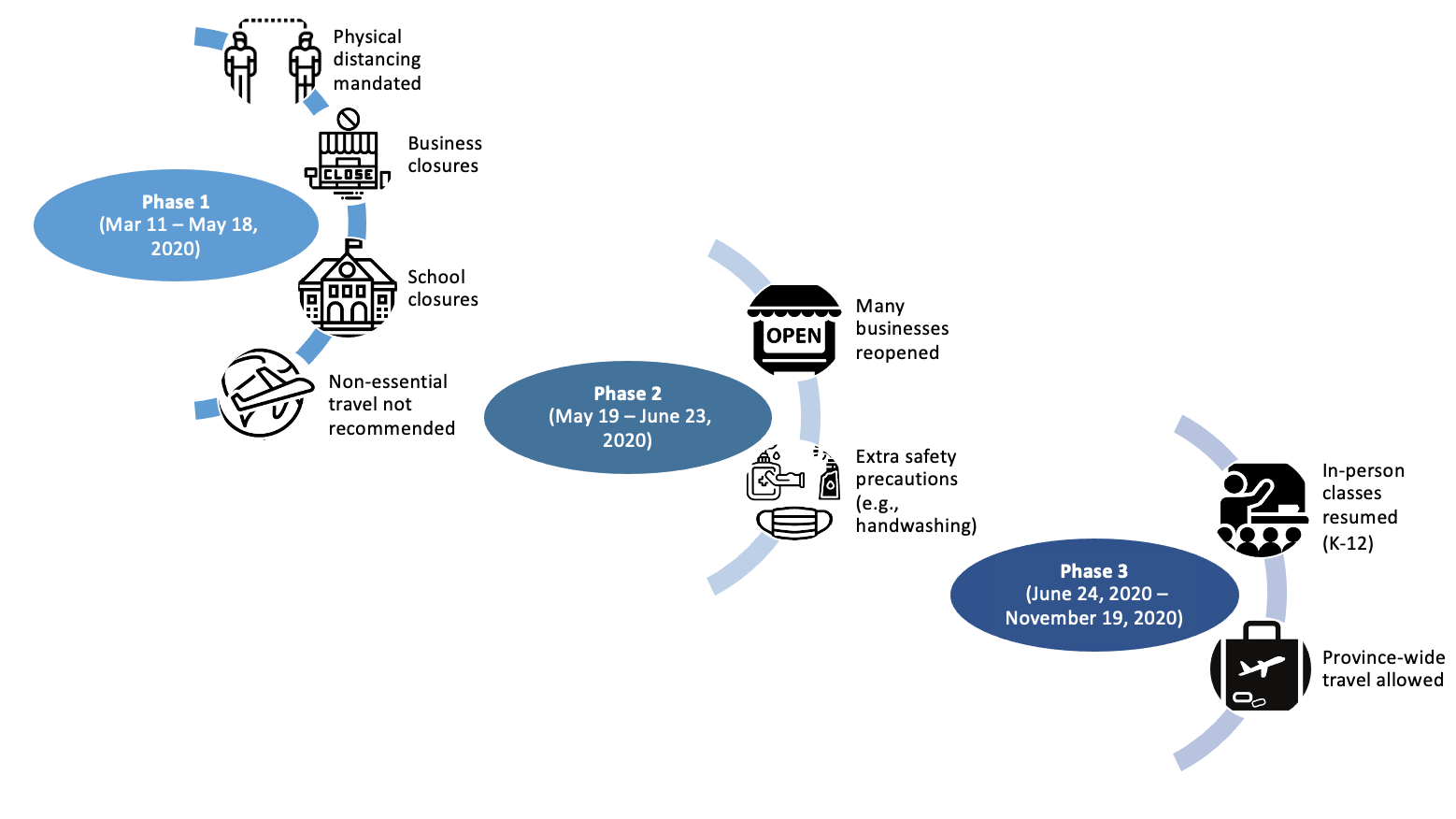
**

Supplement: Supplementary file 1 — Supporting information. [file HEX-25--s002.docx]
