# Supplementary material for: Experiences of self‐care during the COVID‐19 pandemic among individuals with rheumatoid arthritis: A qualitative study
Source: Health Expect. 2021 Aug 17;25(2):482–98. doi: 10.1111/hex.13341 (PMC8444741; doi:10.1111/hex.13341)
Supplement: Supplementary file 2 — Supporting information. [file HEX-25--s004.docx]

**Supplementary File 2: Interview Guide**

**Preamble:** *Thank you for agreeing to take part in this study. The purpose of today’s interview is to understand your experiences of self-care while using the OPERAS website with the physiotherapist in this study. Although we have an interview guide with some questions for you, you may raise any issues which you feel are important. As well as any good days you may have experienced during the pandemic, I’m also interested in hearing about any bad days you might have had. We need to hear about the bad days as well so we can help find the solutions. Please don’t hold back if you’re comfortable sharing that. We will be audio-taping our interview today. Are you okay with proceeding?*

**General Experience:**

1. **Can you talk me through your experience of being involved in this study?**

***Prompts and probes to be used during active listening if appropriate:***

- - How easy or difficult was it for you to be involved in this study?
  - What prompted you to decide to take part?
  - How active were you before the study, compared to during the study?

1. **How has day-to-day life changed for you during this COVID period?**
   - Have you been noticing any changes
     1. At home? (to get at impact on family/social life/leisure)
     2. In your work/education/everyday expenses?
     3. In your diet? (e.g., alcohol or other recreational drugs?)
     4. In your symptoms (pain/stiffness in joints, tiredness, memory loss, anything else)?
     5. In follow-up appointments with health professionals?
     6. Related to the medications/treatments you might take?
        - Access to meds (i.e. 1 vs. 3 month supply of HCQ)
        - Picking up meds
        - Stopping any meds temporarily if you felt you had COVID-19 symptoms (did you decide on this yourself vs. at the guidance of your rheumatologist?)
        - Prescription costs/coverage
   - How have you been feeling?
     - - What about your emotional or mental health (e.g., grief, loss, anxiety, depression, fear, “meltdowns”, “pity party”, psychological/emotional exhaustion, guilt)? Do you anticipate any changes during colder months?
   - What/who helps? Any situations when nothing has seemed to help?
     - - Any particular resources?
   - How easy or difficult has it been for physical activity to be part of your day?
     - - Change in frequency/type/intensity
       - Is your motivation affected? If so, what’s leading it to be affected?
2. **Can you talk me through how you were using the OPERAS website before and during the COVID period?**
   - How easy or difficult was it to use the new OPERAS website?
   - What was the most useful feature? Please tell me more…
   - What was your least favorite feature of the new OPERAS website? Please tell me more…
   - What part of the OPERAS website was most difficult to navigate and why?
   - What part of the OPERAS website was easiest to navigate and why?
   - Has the information changed the way you think about your physical activity/how you feel?
3. **Who else (if anyone) have you talked with about information from the OPERAS website?**

***Prompts and probes to be used during active listening if appropriate:***

- - - E.g., friend, family, colleague, personal trainer, health professional?
    - What prompted you to share the information from the OPERAS website with others?
    - How comfortable or uncomfortable were you sharing the information from the OPERAS website with others?
  - Is there anyone else you can think of?

1. **I would like to hear your thoughts about your experiences with the study physiotherapist before and during the COVID period.**

- Tell me about the in-person counselling session and the physical activity goals set by you and your study physiotherapist
  - Were these goals realistic for you? How did you go about reaching these goals? Example?
  - How easy or difficult was to reach these goals?
- How did the telephone calls go?
  - What did you like/dislike about the sessions? What could have been improved?
- What other interactions did you have with the study physiotherapist?
  - How did these interactions compare to the phone calls?
- Was there anything about the sessions that could have been improved?
- Probe for issues of: trust, responsibility, respect, partnership
- Anything else you would like to add?

1. **Would you recommend the OPERAS website to other people living with RA? Please tell me more…**
   - What advice if any would you give to others who may want to take part in the study?
   - Is there anything else you can think of?
