# Supplementary material for: Experiences of self‐care during the COVID‐19 pandemic among individuals with rheumatoid arthritis: A qualitative study
Source: Health Expect. 2021 Aug 17;25(2):482–98. doi: 10.1111/hex.13341 (PMC8444741; doi:10.1111/hex.13341)
Supplement: Supplementary file 3 — Supporting information. [file HEX-25--s001.docx]

**Supplementary File 3: Patient Partner Involvement in Methods**

**PREPARATORY PHASE:**

- **Identifying and prioritizing research questions**
- Researchers and patient partners in Canada refine the research objective in discussion over email.

**EXECUTION PHASE:**

- **Study designing**
- **Undertaking**
- Patient partners in Canada reviewed and provided feedback via email on a manuscript publication of findings from the study.

**TRANSLATION PHASE**

- **Disseminating**
- Patient partners in Canada advise researchers in developing COVID-19 related questions to modify the interview guide in meetings via Zoom and over email.
- In meetings via Zoom, researchers and patient partners in Canada and the UK discussed themes and patterns identified in the data to inform ongoing analysis and interpretation.

**Dissemination**

**Analysing and**

**Interpreting Data**
